# Research on named entity recognition of adverse drug reactions based on NLP and deep learning

**DOI:** 10.3389/fphar.2023.1121796

**Published:** 2023-06-01

**Authors:** Jianxiang Wei, Tianling Hu, Jimin Dai, Ziren Wang, Pu Han, Weidong Huang

**Affiliations:** ^1^ School of Management, Nanjing University of Posts and Telecommunications, Nanjing, China; ^2^ School of Cyber Science and Engineering, Southeast University, Nanjing, China; ^3^ School of Internet of Things, Nanjing University of Posts and Telecommunications, Nanjing, China; ^4^ School of Computer Science, Nanjing University of Posts and Telecommunications, Nanjing, China; ^5^ Key Research Base of Philosophy and Social Sciences in Jiangsu-Information Industry Integration Innovation and Emergency Management Research Center, Nanjing, China

**Keywords:** named entity recognition, natural language processing, adverse drug reactions, deep learning, information extraction

## Abstract

**Introduction:** Adverse drug reactions (ADR) are directly related to public health and become the focus of public and media attention. At present, a large number of ADR events have been reported on the Internet, but the mining and utilization of such information resources is insufficient. Named entity recognition (NER) is the basic work of many natural language processing (NLP) tasks, which aims to identify entities with specific meanings from natural language texts.

**Methods:** In order to identify entities from ADR event data resources more effectively, so as to provide valuable health knowledge for people, this paper introduces ALBERT in the input presentation layer on the basis of the classic BiLSTM-CRF model, and proposes a method of ADR named entity recognition based on the ALBERT-BiLSTM-CRF model. The textual information about ADR on the website “Chinese medical information query platform” (https://www.dayi.org.cn) was collected by the crawler and used as research data, and the BIO method was used to label three types of entities: drug name (DRN), drug component (COM), and adverse drug reactions (ADR) to build a corpus. Then, the words were mapped to the word vector by using the ALBERT module to obtain the character level semantic information, the context coding was performed by the BiLSTM module, and the label decoding was using the CRF module to predict the real label.

**Results:** Based on the constructed corpus, experimental comparisons were made with two classical models, namely, BiLSTM-CRF and BERT-BiLSTM-CRF. The experimental results show that the *F*_1_ of our method is 91.19% on the whole, which is 1.5% and 1.37% higher than the other two models respectively, and the performance of recognition of three types of entities is significantly improved, which proves the superiority of this method.

**Discussion:** The method proposed can be used effectively in NER from ADR information on the Internet, which provides a basis for the extraction of drug-related entity relationships and the construction of knowledge graph, thus playing a role in practical health systems such as intelligent diagnosis, risk reasoning and automatic question answering.

## 1 Introduction

Adverse Drug Reaction (ADR), mainly refers to a harmful reaction unrelated to the purpose of the medication caused by a qualified drug in normal dosage (Edwards and Aronson, 2000). Since the 1950s, the number of new drugs in the world has increased dramatically, and there are tens of thousands of drug varieties. With the increase in the variety of drugs, the probability of adverse drug events has also increased dramatically. There have been a number of serious adverse drug reactions in the world, for example: in the 1950s and 1960s, thalidomide (Reactive Stop), used for the treatment of morning sickness in pregnancy, caused “malformations in seal-limbed babies,” with a total of more than 10,000 cases and distributed in 17 countries (Zhang et al, 2019); In the early 1980s, the diet pill fenfluramine, which was popular for its weight loss benefits, had serious adverse effects such as heart valve hypertrophy and arrhythmias, damaging the heart valves of hundreds of thousands of dieters in the United States alone (Li, 2001). Due to the small test samples, inadequate observation time and insufficient scope of testing in early clinical trials of medicines, many potential adverse reactions aren’t detected early, leading to risks in the later use of medicines. According to the ADR monitoring report: in 2021, China increased the number of new and serious adverse drug reaction events, reports 597,000, of which serious adverse drug reactions accounted for 11%, posing a great threat to people’s lives (National Medical Products Administration of China, 2022). With the advent of the era of big data, a number of professional medical websites, pharmacovigilance departments, health communities, and other online resources provide a database for drug risk recognition. How to use text mining technology to obtain valuable knowledge from these information resources, so as to provide decision-making services for the safe use of medicines has become an important issue that needs to be addressed urgently. The premise of text mining is to identify the names of drug-related entities such as drug names, drug components and adverse drug reactions from the vast amount of web information.

At a time when computer data is exploding, there is a lot of valuable information hidden in big data, but most of this data is unstructured text that cannot be used directly by computers, and cannot be exchanged in databases, resulting in the dilemma of “abundant information but lack of knowledge,” Based on this problem, natural language processing tasks have arisen intending to enable computers to understand the unstructured text and actively learn through various methods and techniques to access information in the unstructured text (Xi and Zhou, 2016; DWOtterMedina and Kalita, 2020). Named entity recognition, refers to the recognition of entities with a specific meaning in natural language text (Nadeau and Sekine, 2007). The named entity recognition task simply means extracting keywords or information from text information, for example, recognizing entities such as the name of a person, place, time, etc. present in a piece of text. The result of named entity recognition includes the type and boundary of that named entity. Named entity recognition is an important stage in the process of moving from theoretical to practical applications of natural language processing techniques, and it generally underpins many natural language processing tasks, for example, the recognition of named entities associated with a graph needs to be implemented before a knowledge graph can be constructed using text.

Named entity recognition techniques can help people understand unstructured texts about adverse drug reactions, and also prepare the groundwork for subsequent research on entity relationship extraction and knowledge graph construction. To this end, this paper proposes a named entity recognition method for adverse drug reactions based on the ALBERT-BiLSTM-CRF model. Among them, the method adopts BIO annotation model for corpus characteristics and extracts three types of named entities based on ALBERT-BiLSTM-CRF model for drug names, drug components and adverse drug reactions, so as to mine the knowledge related to adverse drug reactions and help people understand the risks of drugs.

## 2 Related work

Named entity recognition is initially proposed as a sub-topic of information extraction and has evolved to the point where the main approaches to named entity recognition are classified as lexicon and rule-based, statistical machine learning-based and deep learning-based. Lexicon and rule-based methods are the first named entity recognition methods proposed, and currently, deep learning-based named entity recognition methods have become mainstream methods (Li et al, 2020).

Deep learning is a collection of algorithms that apply machine learning to multilayer neural networks. Deep learning is more adaptable to data and can acquire features from the raw data itself, reducing human costs and the impact of subjectivity. Common deep learning network structures include: Recurrent Neural Network (RNN), Long Short-Term Memory (LSTM) and Gated Recurrent Unit (GRU) (Chiu and Nichols, 2016; Dauphin et al, 2017; Strubell et al, 2017; Zhao et al, 2019).

In order to parse natural scenes and natural language, scholars proposed the RNN model. Later, the Recurrent Neural Tensor Network (RNTN) model, which is based on the RNN model, was proposed and mainly applied to sentiment analysis.

RNNs suffer from a “long-term dependency problem,” where they can only learn short-term information because the gradient disappears. In view of the problems of RNNs, Hochreiter et al proposed a LSTM model in 1997 (Hochreiter, Schmidhuber, 1997). The LSTM improves the RNN by controlling the rate of accumulation of information through the design of gating, successfully solving the gradient disappearance problem and learning long-range dependencies. The LSTM supports selective addition and selective forgetting, the feature makes the LSTM well-suited for applications in named entity recognition problems (Chen et al, 2015). Hammerton et al first applied LSTM in the area of named entity recognition (Hammerton, 2003).

The LSTM structure only has a forward hidden layer, so it can only handle forward sequence information, while BiLSTM is a Bi-directional LSTM (Graves and Schmidhuber, 2005), a forward LSTM and a backward LSTM, respectively, whose output is obtained by splicing the state vectors of the forward and backward LSTMs, and the two LSTMs are completely independent in encoding, not sharing parameters and state vectors, fully acquiring information and encoding. Lample et al used BiLSTM to encode sequence context information and proved that BiLSTM is better than LSTM (Lample et al, 2016).

Darshini et al explored the relationship between a drug and its associated attributes using three approaches: a rule-based approach, a deep learning-based approach, and a contextualized language model-based approach on the n2c2-2018 ADE extraction dataset. They proved that the contextualized language model-based approach outperformed other models overall (Mahendran and McInnes, 2021). Christopher et al developed a deep learning natural language processing algorithm to identify ADRs in discharge summaries at a single academic hospital centre (McMaster et al, 2023).

This paper chooses to improve on the classical model BiLSTM-CRF, a deep learning recognition method, by introducing the ALBERT model in the input representation layer to achieve the recognition of Chinese adverse drug reaction named entities. Different from the existing approaches, our contributions lie in:

1) We innovatively proposed an ALBERT-BiLSTM -CRF model-based named entity recognition method for adverse drug reactions, which outperforms the BERT-BiLSTM-CRF and BiLSTM-CRF-based methods.
2) We customized the annotated lexicon and constructed a Chinese corpus of adverse drug reactions using the BIO sequence annotation method for training named entity recognition models without laborious manual annotation.
3) The Chinese corpus of adverse drug reactions constructed in this paper contains both Western and Chinese medicines, with more comprehensive data than previous studies.

## 3 Materials

### 3.1 Sources of data

In this paper, the website of “China Pharmaceutical Information Query Platform” (https://www.dayi.org.cn/) was selected as the main data source, and Python crawling technology was used to crawl the data from the website. The data included drug names, drug components and adverse drug reactions. Then we annotate various entities based on these data through manual annotation.

### 3.2 Data pre-processing

The raw data contained 34,064 drug data, which contained duplicate items, null values, garbled codes and data not relevant to the experiment. Data pre-processing of the raw data was required to process the raw data into the format required for the experimental dataset.

The main tasks of data pre-processing were: 1) removing garbled, invisible characters and null data; 2) removing duplicate drug data; and 3) dividing the initially processed data in such a way that one TXT file was generated for each drug data. A total of 12,977 TXT files were eventually generated, with each TXT file being the data for one drug. The average length of the data for one drug is 148 Chinese characters. The purpose of this is to facilitate the debugging of subsequent programs, reduce the amount of single processing, and provide a more intuitive view of the entity recognition effect.

### 3.3 Customized annotation dictionaries

In this paper, three types of entities, namely, drug name (DRN), drug component (COM) and adverse drug reaction (ADR), were defined for the definition requirements of the adverse drug reaction knowledge graph and the experimental data format. In this experiment, the deep learning corpus was prepared by using BIO sequence annotation, and the annotation was performed automatically according to the entity words and entity labels in the dictionary. The matching of drug names, drug components and the presentation of adverse drug reactions is therefore largely determined by the completeness and accuracy of the lexicon entries. The experiments in this study required an annotated lexicon consisting of drug name, drug component and adverse drug reaction entries.

Named entity recognition has been widely studied in different languages and different domains, but a large number of related studies are based on already existing corpora. The existing corpora are mainly English corpora or based on classical named entities, such as personal names and place names, although there are a small number of corpora focusing on other entities, they are still some distance away from the adverse drug reaction entities studied in this paper.

As there is no authoritative corpus available, this study used a customized dictionary approach to write the collected drug names, drug components and adverse drug reaction entity entries into a dictionary, stored each entry in one row and added the entity type to which each entry belongs to construct the dictionary used for this experimental BIO annotation corpus. Two different sources for the collection of relevant lexical entries are shown in Table 1.

**TABLE 1:** Sources of terms related to drug components and adverse reactions.

Source	Web link
Baidu Input Method Lexicon	https://shurufa. baidu. com/dict
Sogou Input Method lexicon	https://pinyin. sogou. com/dict/

Although the lexicon shared by the input method may include incorrect entries (Gong, 2019), such as the inclusion of Chinese herbal prescriptions in drug components, both input method lexicons were chosen as sources for this paper, considering that the priority of building a lexicon is to ensure that the number of entries collected is large enough and that the lexicon shared by the input method contains a large number of entries uploaded by different authors or institutions with rich data content. Moreover, in order to include as many entries as possible, all lexicons files on drug names, drug components and adverse drug reactions were used.

As multiple lexicons files were used previously to ensure that the collection was large enough, but this may have included the same entries, for example, lexicons file a and lexicons file b. Although file b complements a, some entries are the same as file a, so duplicate entries need to be removed. This step is implemented by Python programming, the specific idea is: 1) read the contents of the lexicons file by line; 2) store the read contents in a set type variable. Duplicate entries are removed by using the property that set type variables do not contain duplicate elements.

In the end, a total of 12,977 entries were collected for drug names, 16,308 entries for drug components and 11,141 entries for adverse drug reactions. The statistics on the number of entities in the lexicons are shown in Table 2.

**TABLE 2:** Type and number of entries.

Type of entity	Quantity	Example
DRN	12,977	Allopurinol Tablets
COM	16,308	Allopurinol
ADR	11,141	Skin rash

### 3.4 Corpus construction

The model used for the experiments in this study is the ALBERT-BiLSTM-CRF model, and preparing a corpus for model training is an essential task before training this model. The corpus requires that each word in the corpus is followed by a label for that word, by which it is possible to indicate whether the word being labeled belongs to the target named entity and if so, also whether it is the first word or one of the middle words of the target named entity. If more than one category of named entity is to be identified at the same time for this named entity recognition task, multiple entity labels are to be used.

Sequence labeling is a fundamental problem in NLP problems. In sequence annotation, we want to label each element of a sequence. In general, a sequence refers to a sentence, while an element refers to a word in a sentence. There are two commonly used sequence annotation methods: BIO annotation and BIOES annotation. The corpus of this study used the BIO sequence annotation model to annotate entities in sentences.

After BIO annotation of data, each record in the corpus should include two fields: 1) the text of the drug name, drug component and adverse reaction entity; 2) the BIO label sequence corresponding to that text.

The generation of BIO sequence annotation was done by Python programming, based on the customized annotation dictionaries built in Section 3.3. The specific process was: 1) according to the constructed annotation dictionary, to annotate the text to be keyword matching, a successful match will be marked with the corresponding entity label, not a successful match will be marked as “O,” the specific entity label is defined in Table 3 below; 2) the BIO annotated corpus is written to a text file, with a blank line as a separating mark between sentences. Entity label definitions is shown in Table 3.

**TABLE 3:** Entity label definitions.

Type of entity	Start label	Middle label
DRN	B-DRN	I-DRN
COM	B-COM	I-COM
ADR	B-ADR	I-ADR

The self-built corpus contains 12,977 records. Each word in each record is followed by a label to form a row of data separated by spaces, i.e., “word O\ B-(DRN, COM, ADR),” e.g., “Bifenprox tablets: bifenprox. Mild nausea and occasional skin rash may occur in some cases.” The Chinese labeling example is shown in Table 4.

**TABLE 4:** Example of adverse drug reaction corpus annotation.

Word	Label	Word	Label	Word	Label
联	B-DRN	个	O	轻	O
苯	I-DRN	别	O	度	O
双	I-DRN	病	O	恶	B-ADR
酯	I-DRN	例	O	心	I-ADR
片	I-DRN	服	O	,	O
:	O	用	O	偶	O
联	B-COM	后	O	有	O
苯	I-COM	可	O	皮	B-ADR
双	I-COM	出	O	疹	I-ADR
酯	I-COM	现	O	。	O

At this point, the corpus has been prepared for the experimental simulation, after which the corpus was divided into the training set, validation set, and test set and passed into the ALBERT-BiLSTM-CRF model for experimental simulation.

## 4 Materials and methods

### 4.1 Model framework

**FIGURE 1:**
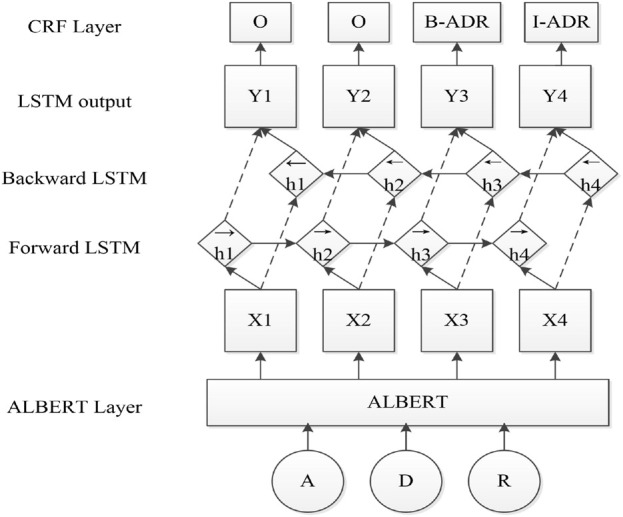
Model framework of ALBERT-BiLSTM-CRF.

Based on the superiority of the ALBERT pre-training model, this paper implements named entity recognition of adverse drug reactions based on the ALBERT-BiLSTM-CRF model, which consists of three main components: the ALBERT pre-training model, the BiLSTM layer and the CRF layer. These three parts are used in the input representation layer, sequence modeling layer and prediction decoding layer in turn, and the main structure of the model is shown in Figure 1.

The model consists of three parts: 1) The input representation layer, where the ALBERT module maps each word into a word vector through the Embedding layer and then uses the Transformer layer to obtain character-level semantic information, specifically by encoding contextual features after a two-way synthesis and adding the learned syntactic and semantic level information to the token. 2) The Context Encoder layer, which inputs the obtained word vectors into the BiLSTM module, performs high-dimensional feature extraction based on the contextual information. 3) Label decoder layer, the classical model CRF is used in this layer to predict the real label sequence.

**FIGURE 2:**
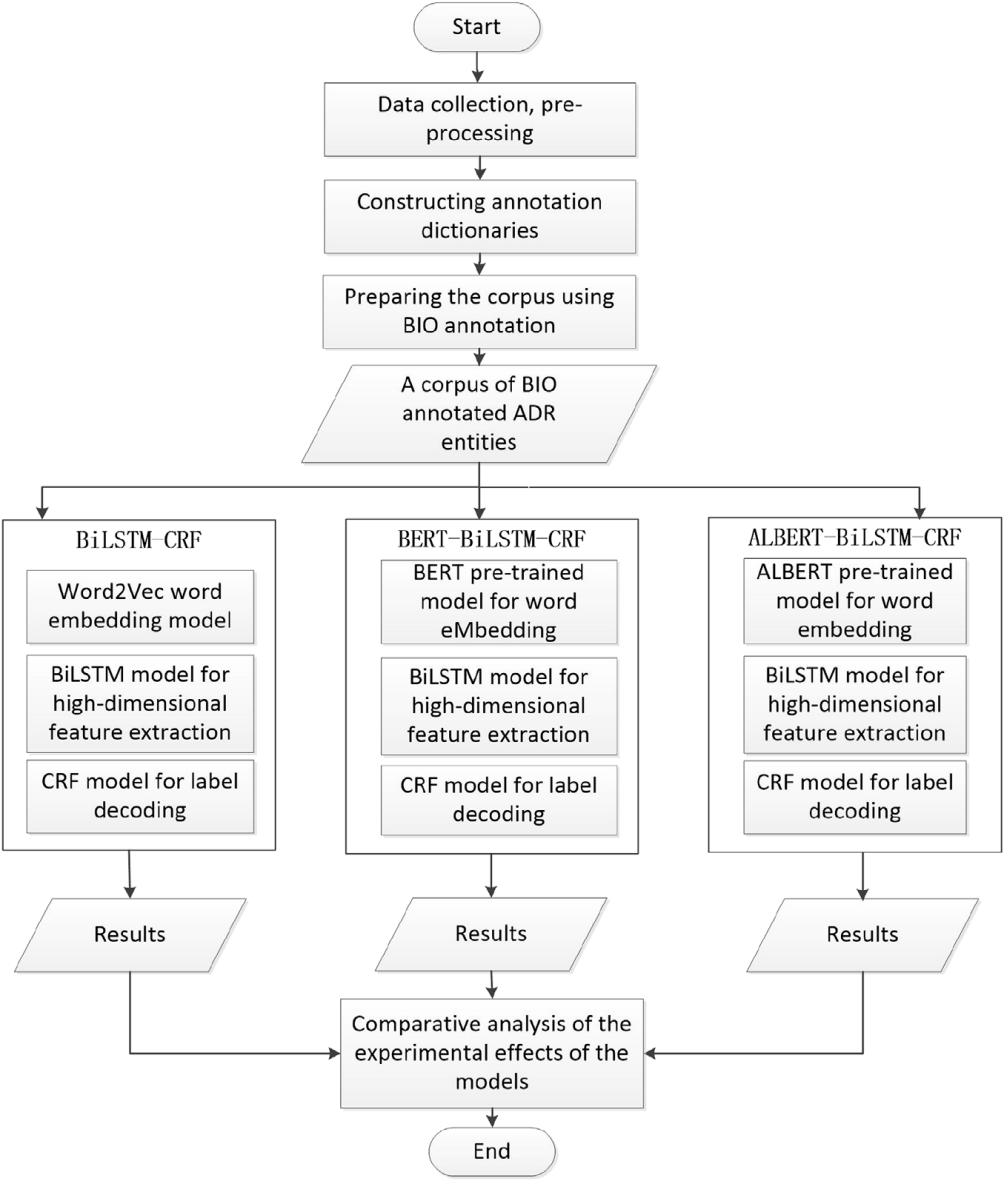
Flow chart of the method.

In this paper, an ALBERT model was introduced in the input representation layer of the classical BiLSTM-CRF model, and an ALBERT-BiLSTM-CRF model-based named entity recognition method for adverse drug reactions was proposed. The method used the BIO approach to annotate three types of entities, namely, drug name (DRN), drug component (COM) and adverse drug reaction (ADR) for the corpus characteristics of drug instructions, and extracted these three types of entities by using the ALBERT-BiLSTM-CRF model. In this paper, the BiLSTM-CRF and BERT-BiLSTM-CRF models were selected as control models for experimental simulation with precision P, recall R and *F*_1_ score as performance evaluation indicators. By comparing and analyzing the evaluation metrics of each model, the superiority of applying the ALBERT-BiLSTM-CRF model to the adverse drug reaction named entity recognition task was demonstrated, and the specific implementation flow is shown in Figure 2.

### 4.2 ALBERT pre-training model

The named entity recognition task consists of three main components: the distributed representation of the input, the context encoder and the label decoder (Chiu and Nichols, 2016). The distributed representation of the input task implements the encoding of the input word into a vector of real numbers, and the context encoder and label decoder process the real vector and, through mapping, confirm the entity label of the word.

The earliest approach to the distributed representation of input is the one-hot, which later give rise to methods such as the bag-of-words model and the n-gram. In order to take into account the relationships between word vectors, the distributed representation of the input has evolved to the currently dominant Word2Vec embedding method (Mikolov et al, 2013). Although it addresses some contextual issues, Word2Vec only provides a layer of representation and does not yet address the issue of word polysemy. The pre-training model BERT uses the Encoder part of the bidirectional Transformer (Vaswani et al, 2017) to compute the relationship between input and output, and structures such as RNN/CNN are completely discarded by BERT and the Attention mechanism takes its place. With this improvement, BERT makes full use of contextual information and defines different vector representations for words based on different contexts, solving the problem of multiple-meaning words. However, the high performance of the BERT model relies on the introduction of more parameters, which increases the complexity of the model, and the training time of the BERT model increases significantly and requires more hardware.

Because of the problems of BERT, Lan et al (Lan et al., 2019) proposed a lightweight BERT-ALBERT. ALBERT hardly changes the model architecture of BERT, but ALBERT significantly reduces the number of parameters, accelerates the training speed and overcomes the problem of difficulty in extending the model without affecting its performance.

**FIGURE 3:**
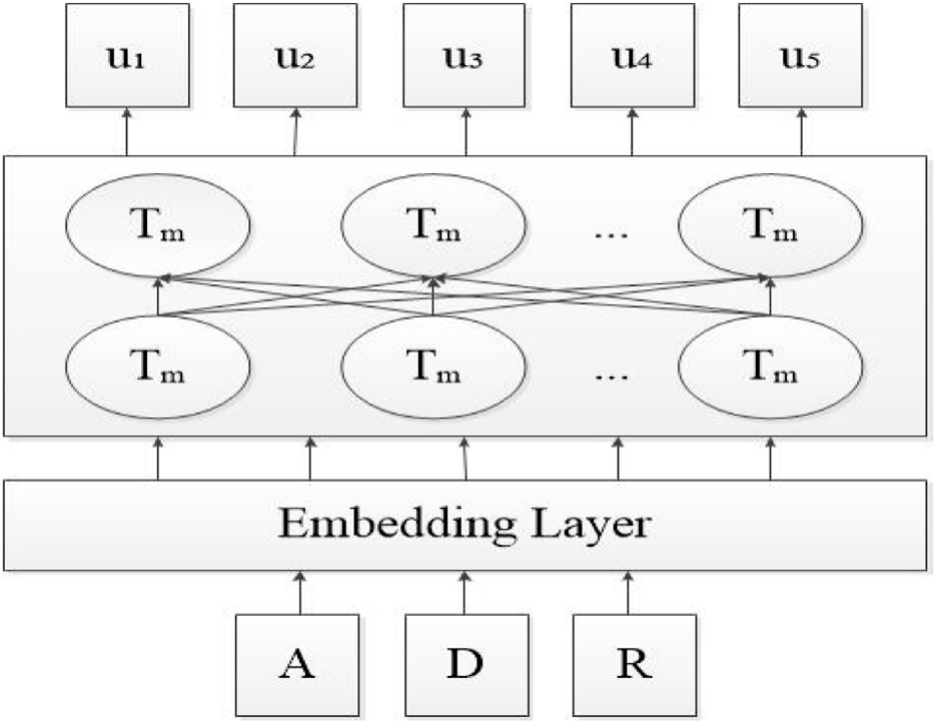
Structure of the ALBERT pre-training model.

ALBERT reduces the parameters by embedding the matrix decomposition technique and cross-layer parameter sharing mechanism, discarding the next sentence prediction (NSP) training method and proposing sentence order prediction (SOP) training method instead, thus improving the performance of downstream tasks. The structure of the ALBERT pre-training model is shown in Figure 3.

As can be seen from Figure 3, the structure of the ALBERT pre-training model mainly consists of an Embedding Layer and a Transformer encoder layer (Ma and Huang, 2021). The input corpus words are transformed into a vector through the Embedding Layer, and the word vector dimension can be taken as 128 or 300, after which the Embedding Layer feeds the processed vector to the encoder layer.

The encoder layer is the encoder part of the Transformer and is built from a multi-headed Self-attention and fully-connected layer. The Self-attention is calculated as follows:

$$Attention\left(Q,K,V\right)=softmax\left(\frac{QK^{T}}{\sqrt{d_{k}}}\right)V$$ (1)

In which, Q,K,V are the 3 input matrices obtained by linear transformation of the input vectors, and $d_{k}$ is the input word vector dimension. Different weights are obtained by calculating the magnitude of the relational weight between each input word vector and the other word vectors of the sequence, and then the weights are weighted and summed with the representations of all the sequences to finally obtain the new character representations.

To train the encoder layer, ALBERT uses mask learning and SOP. The SOP task is to determine whether two sentences are adjacent to each other in the original text, as well as the order and coherence of the sentences, and was proposed to improve the NSP of the BERT model. Many studies have shown that the NSP originally designed to improve the performance of downstream tasks is not very efficient, so ALBERT proposed SOP, and the experimental results show that SOP can solve the NSP inefficiency problem within a reasonable range.

### 4.3 BiLSTM

BiLSTM takes up the work of the context encoder in the named entity recognition task. This paper used the ALBERT-BiLSTM-CRF model for named entity recognition of adverse drug reaction entities, in which sequence features in the text of adverse drug reactions were obtained by BiLSTM. BiLSTM used the LSTM (Chen et al, 2015) structure in the implicit layer unit.

The LSTM has been improved to avoid the problem of gradient disappearance and has a longer memory in response to the shortcomings and problems of the RNN model. The LSTM avoids gradient disappearance mainly through the transmission band, which is the most important structure of the LSTM model.

The LSTM consists of four parts: oblivion gate $f_{t}$ , input gate $i_{t}$ , transmission band $C_{t}$ and output gate. The oblivion gate $f_{t}$ takes a value in the middle of 0–1. If an element of $f_{t}$ exists that is 0, the value of the transmission band $C_{t-1}$ cannot be passed, achieving selective forgetting, while the input gate $i_{t}$ adds information to the transmission band C , and by doing so, information is updated. It is this selective recording and selective forgetting that avoids the gradient explosion and disappearance problems and distance dependence of RNNs.

The whole process is as follows.

$$f_{t}=\sigma \left[W_{f}\cdot \left[h_{t-1},X_{t}\right]+b_{f}\right]$$ (2)

$$i_{t}=\sigma \left[W_{i}\cdot \left[h_{t-1},X_{t}\right]+b_{i}\right]$$ (3)

$${\tilde{C}}_{t}=\tanh\left[W_{C}\cdot \left[h_{t-1},X_{t}\right]+b_{C}\right]$$ (4)

$$C_{t}=C_{t-1}\circ f_{t}+i_{t}\circ {\tilde{C}}_{t}$$ (5)

$$o_{t}=\sigma \left[W_{o}\cdot \left[h_{t-1},X_{t}\right]+b_{o}\right]$$ (6)

$$h_{t}=o_{t}\circ \tanh\left[C_{t}\right]$$ (7)

The meanings of the variables in the formula are shown in Table 5.

**TABLE 5:** Meaning of variables.

Variables	Meaning (at moment t)	Variables	Meaning (at moment t)
σ	Sigmoid activation function	$h_{t}$	State vectors
${\tilde{C}}_{t}$	Intermediate states	$X_{t}$	Input vectors
$f_{t}$	Oblivion gate	b	Bias vector
$i_{t}$	Input gate	tanh	Hyperbolic tangent function
$C_{t}$	Transmission band	W	Model parameter matrix
$o_{t}$	Output gate		

The BiLSTM used in this paper is a Bi-directional LSTM whose output is obtained by splicing the state vectors of the forward and backward LSTMs, and the two LSTMs are completely independent in their encoding and do not share parameters or state vectors.

### 4.4 CRF

The BiLSTM takes on the work of the context encoder in the named entity recognition task, while the CRF model is the label decoder, predicting the output sequence from the input sequence. Given a set of observed sequences $X=\left\{x_{1},x_{2},\cdots ,x_{n}\right\}$ , the predicted sequence labels $y=\left\{y_{1},y_{2},\cdots ,y_{n}\right\}$ can be obtained.

In named entity recognition, after mapping each character into a word vector and taking into account the context, the output of the BiLSTM is a score indicating that each word corresponds to each entity category, and the category label with the highest score can be selected as the predicted result. However, there is a problem with such named entity recognition in that BiLSTM cannot restrict the relationship between the two labels before and after, and the output results do not affect each other, simply selecting the label with the highest score at each step as the output label. For example, in the BIO labeling process, if “B-entity type” is used to denote the beginning of an entity and “I-entity type” to denote the middle part of an entity, then a sequence of {I-entity type 1, I-entity category 2} must be wrong.

In view of this problem, in 2001, Lafferty et al first proposed a CRF for the sequence annotation problem, which has a feature transfer matrix, based on which the CRF can learn some constraints on the labels during training, such as “no O-entity type” (Lafferty et al, 2002).

The score for the label y corresponding to the text X is calculated as follows.

$$score\left(X,y\right)=\sum_{i=1}^{n}P_{i,y_{i}}+\sum_{i=0}^{n}A_{y_{i},y_{i}+1}$$ (8)

In the formula, $A_{y_{i},y_{i}+1}$ denotes the transfer fraction from label $y_{i}$ to label $y_{i+1}$ ; $p_{i,y_{i}}$ is the emission matrix, indicating the fraction of the ith character predicted to be labeled $y_{i}$ , and the fraction of label y corresponding to text X is the sum of the transfer matrix A and the emission matrix P .

To maximize the probability of a correct sequence, the CRF is given a linear chain of conditional random fields $P\left(y|X\right)$

$$P\left(y|X\right)=\frac{\exp\left(score\left(X,y\right)\right)}{\sum_{y^{\prime }}\exp\left(score\left(X,y^{\prime }\right)\right)}$$ (9)

In the formula, y is the true sequence and $y^{\prime }$ is the set of all possible sequences.

### 4.5 Experimental design

#### 4.5.1 Experimental environment

This experiment was conducted on Ubuntu 20.04.3 LTS, with a GPU version of RTX 2080 Ti with 11G of video memory, a 12-core Intel(R) Xeon(R) Platinum 8255C CPU @ 2.50GHz, 45G of system memory, a Tensorflow version of 1.15.0 GPU, and the language used Python 3.6.

#### 4.5.2 Parameter setting

The experimental data source is a self-built corpus, the specific construction process of which is described in Section 3. The data content is mainly a corpus of drug information on adverse drug reactions, containing drug names, drug components and possible adverse reactions caused by drugs, with a total of 12,977 records. Ultimately, this paper divided the corpus into three parts with a ratio of 6:2:2, which are used as the training set, the validation set and the test set. The training set is used to train the neural network model, and then the validation set is used to verify the validity of the model, selecting the model that gives the best results, until we have a satisfactory model. Finally, once the model has passed the validation set, this paper then uses the test set to test the final effect of the model. We use the bert-base model and albert-base model pretrained in the hugging face to fine-tune our NER task.

The BiLSTM-CRF and BERT-BiLSTM-CRF models were selected as the control group for experimental comparison and analysis to verify the performance of the ALBERT-BiLSTM-CRF model. The experiments used the ALBERT_BASE model released by Google, which has an Embedding of 128, a hidden layer number of 12, a hidden layer dimension of 768, a Layers value of 12 and used gelu, with an overall parametric number of only 12M. The number of hidden layer nodes of BiLSTM was 100. The training parameters of the ALBERT-BiLSTM-CRF model are listed in Table 6. In addition, the comparison experiments used the BERT_BASE model with an Embedding of 128, a hidden layer count of 12, a hidden layer dimension of 768, a Layers value of 12, and gelu with an overall parametric count of 110M.

**TABLE 6:** Training parameters of ALBERT-BiLSTM-CRF model.

Parameter category	Value
Maximum sequence length	128
ALBERT Learning Rate	5e-5
Other module learning rates	0.001
Dropout	0.5
Batch_size	128
epoch	7

**FIGURE 4:**
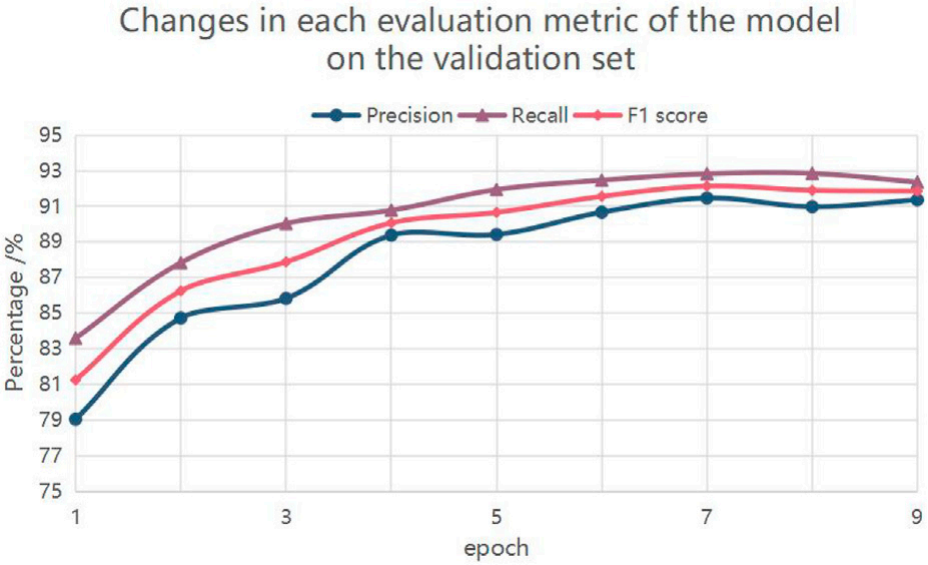
Changes in each evaluation metric of the model on the validation set.

The experiments were set up with different epoch values to study the fitting condition of the model with the number of iterations, so as to determine the appropriate epoch value. The experimental results are shown in Figure 4, where the horizontal coordinates are the epoch values and the vertical coordinates are the percentage performance of entity recognition. The dashes in the graph show the changes in *F*_1_, accuracy and recall respectively. As can be seen from the figure, the values of *F*_1_ and accuracy rate are 92.14% and 91.47% respectively at the 7th epoch, reaching the highest; the recall rate reaches the optimal value of 92.85% at the 8th epoch. With the increase of training times, the model was gradually fitted and converged to a steady state. Taking into account, 7 was chosen as the epoch value for the experiment in this study.

#### 4.5.3 Evaluation metrics

The method was evaluated by using some experimental performance metrics: precision P, recall R and *F*_1_ score as follows.

$$P=\frac{TP}{TP+FP}$$ (10)

$$R=\frac{TP}{TP+FN}$$ (11)

$$F_{1}=\frac{2\mathrm{PR}}{P+R}$$ (12)

The meanings of the variables are shown in Table 7.

**TABLE 7:** Meaning of variables.

Variables	Meaning
TP	Real entities correctly recognized by the model
FP	Real entities not recognized by the model
TN	Non-entities correctly recognized by the model
FN	Non-entities incorrectly recognized by the model

To make the meaning clearer, an example of an ADR entity, “rash,” is given in this paper. Named entity recognition usually identifies more than one type of entity at a time, so for convenience here it is assumed that ADR entities are identified individually. For the entity “rash,” there are 2 possible predicted outcomes: ADR entity and non-ADR entity. If the rash is identified as an ADR entity by the model, the result is called TP ; if the rash is identified as a non-ADR entity, the result is called FP ; if the non-ADR entity is identified as a non-ADR entity, the result is called TN ; if the non-ADR entity is identified as an ADR entity, the result is called FN .

## 5 Results

### 5.1 Model performance evaluation results

In terms of model performance evaluation metrics, this study compared the validation set and test set results of the three models. After 7 epochs, the specific training results of the ALBERT-BiLSTM-CRF model, the BiLSTM-CRF and BERT-BiLSTM-CRF are shown in Table 8.

**TABLE 8:** Comparison of model performance evaluation indicators on different set (%).

Set	Model	Precision	Recall	*F1* score
Validation Set	BiLSTM-CRF	88.54	91.25	89.87
	BERT-BiLSTM-CRF	89.05	92.30	90.64
	ALBERT-BiLSTM-CRF	91.47	92.83	92.14
Test Set	BiLSTM-CRF	88.20	91.23	89.69
	BERT-BiLSTM-CRF	88.02	91.70	89.82
	ALBERT-BiLSTM-CRF	89.88	92.55	91.19

### 5.2 Entity performance evaluation results

Entity evaluation metrics refer to the calculation of precision P, recall R and *F*_1_ score separately for each entity. In terms of entity evaluation metrics, the results of the three types of entity recognition for each model are shown in Table 9.

**TABLE 9:** Entity recognition performance indicators for each model (%).

Evaluation indicators		BiLSTM-CRF	BERT-BiLSTM-CRF	ALBERT-BiLSTM-CRF
Precision	DRN	87.91	83.42	87.78
	COM	89.29	88.96	90.28
	ADR	85.50	87.67	90.09
Recall	DRN	78.90	80.39	80.56
	COM	93.45	93.38	94.42
	ADR	89.86	92.15	92.73
*F*_1_ score	DRN	83.16	81.88	**84.01**
	COM	91.32	91.12	**92.30**
	ADR	87.63	89.86	**91.39**

The bold font is to emphasize the increase in the *F*_1_ score of the ALBERT-BILSTM-CRF model used in this article compared to other models.

**FIGURE 5:**
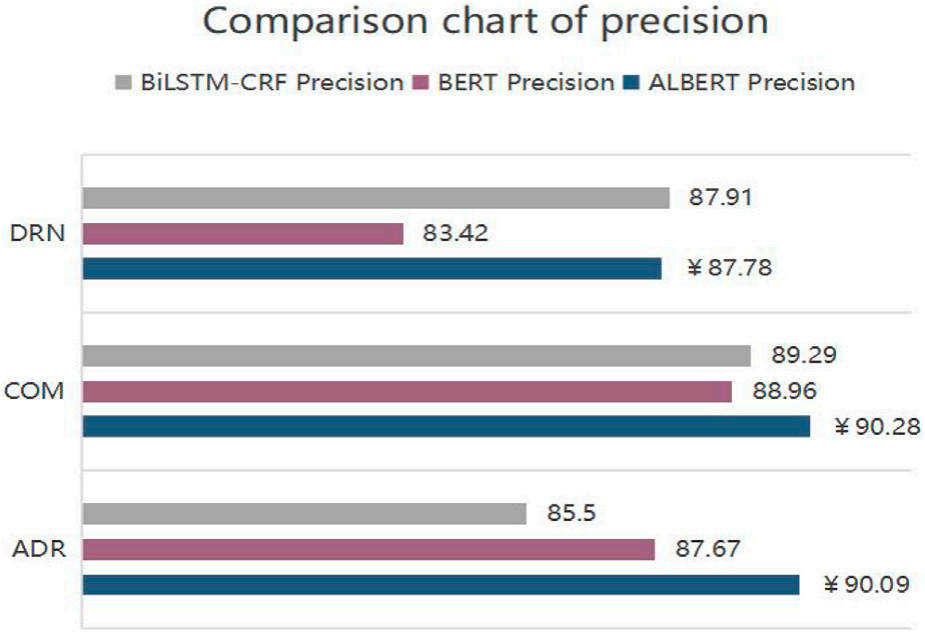
Comparison chart of precision.

**FIGURE 6:**
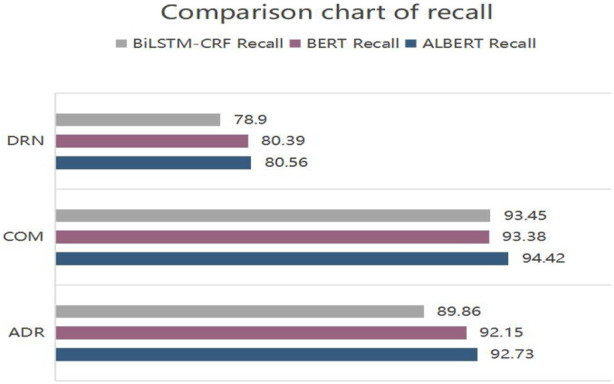
Comparison chart of recall.

**FIGURE 7:**
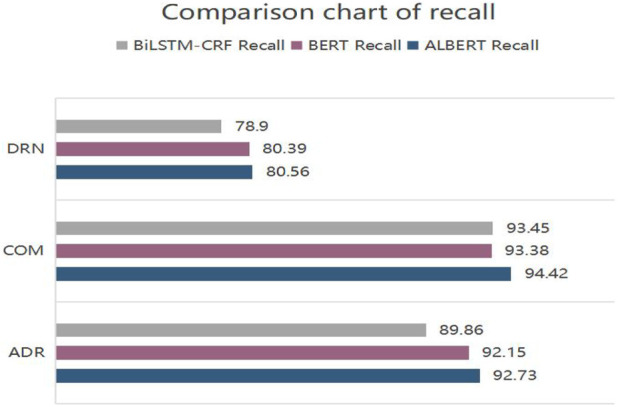
Comparison chart of *F*_1_ score.

Figures 5–7 show the comparison of the experimental results of the models in terms of accuracy, recall and *F*_1_ score respectively.

## 6 Discussion

In this paper, an ALBERT-BiLSTM-CRF model-based named entity recognition method for adverse drug reactions was proposed. The method adopted a BIO approach to annotate three types of entities, namely, drug name (DRN), drug component (COM) and adverse drug reaction (ADR), for the corpus characteristics of drug instructions, and used the ALBERT-BiLSTM-CRF model to extract these three types of entities. In this paper, the BiLSTM-CRF and BERT-BiLSTM-CRF models were selected as the comparison models for experimental simulation with precision, recall and *F*_1_ score as performance evaluation metrics.

As can be seen from Table 8, after 7 epochs, the *F*_1_ score of the BiLSTM-CRF model is 89.69% and the *F*_1_ score of the BERT-BiLSTM-CRF model is 89.82%, an improvement of 0.13%, which indicates that BERT extracts the semantic information of the text better than Word2vec by obtaining word vectors through the bidirectional Transformer. The *F*_1_ score of the ALBERT-BiLSTM-CRF model is 91.19%, which is an improvement of 1.5% and 1.37% over BiLSTM-CRF and BERT-BiLSTM-CRF respectively, which proves the superiority of the method in this paper.

In addition, in terms of training time, it took a total of 31 min to train the BERT-BiLSTM-CRF, while the ALBERT-BiLSTM-CRF model only took 17 min, which is nearly double the speed. The reason for this is that ALBERT reduces the number of parameters, so the number of parameters that need to be updated in the gradient during training is greatly reduced and the training speed is accelerated. In summary, the ALBERT-BiLSTM-CRF model improves training efficiency while maintaining performance, and the method of introducing ALBERT into the model input representation layer solves the problem of redundant parameters in BERT and has research significance.

The analysis in Table 9 shows that the models are least effective in recognizing DRN, more effective in recognizing ADR and best in recognizing COM.

The reason for the poor performance in recognizing drug names (DRN) is that, firstly, drug names contain a large number of parts that are identical to drug components, which can be easily misidentified as drug components when recognizing drug names; secondly, the number of entries for drug names was small when the lexicon was first constructed, which may lead to the poor performance. Secondly, the *F*_1_ score for recognizing ADR is lower than that for recognizing components of COM, both because there are many entries in the corpus where the description of ADR is “unclear” and because there are many statements with the same meaning and different expressions in the description of ADR, for example, “increase” “rise” “improve”. This may lead to inadequate inclusion of ADR in the construction of the lexicon, which may result in the model being less effective in recognizing ADR entities.

The best results were obtained for the recognition of COM, probably because the characteristics of drug components are very obvious, most of the components of western drugs are chemical substances, while most of the components of traditional Chinese medicine are two-word herbs, which are very different from the characteristics of other entities. In addition, the largest number of samples of drug components in this corpus is also more accurate, so the model has the best results for the recognition of COM.

ALBERT-BiLSTM-CRF model is superior to BiLSTM-CRF and BERT-BiLSTM-CRF models in recognizing DRN, COM, and ADR entities. This is demonstrated by the following.

(1) In terms of precision, the precision of DRN on the ALBERT-BiLSTM-CRF model is 87.78%, which is 0.13% less than BiLSTM-CRF and 4.36% better than BERT-BiLSTM-CRF; the precision of recognizing COM is 90.28%, which is 0.99% better than BiLSTM- CRF by 0.99% and BERT-BiLSTM-CRF by 1.32%; the precision for recognizing ADR is 90.09%, an improvement of 4.59% over BiLSTM-CRF and 2.42% over BERT-BiLSTM-CRF. Contrastingly, the precision of ALBERT-BiLSTM-CRF in recognizing each entity has improved significantly.
(2) In terms of recall, the recall for recognizing DRN on the ALBERT-BiLSTM-CRF model is 80.56%, an increase of 1.66% over BiLSTM-CRF and 0.17% over BERT-BiLSTM-CRF; the recall for recognizing COM is 94.42%, an increase of 0.97% over BiLSTM-CRF by 0.97% and 1.04% compared to BERT-BiLSTM-CRF; the recall for recognizing ADR is 92.73%, an increase of 2.87% compared to BiLSTM-CRF and 0.58% compared to BERT-BiLSTM-CRF. The overall comparison also showed a large increase in the recall rate for each entity recognized by ALBERT-BiLSTM-CRF.
(3) In terms of *F*_1_ score, the *F*_1_ score for DRN on the ALBERT-BiLSTM-CRF model is 84.01%, an improvement of 0.85% over BiLSTM-CRF and 2.13% over BERT-BiLSTM-CRF; the *F*_1_ score for recognizing COM is 92.3%, an improvement of The *F*_1_ score for recognizing ADR is 91.39%, an improvement of 3.76% over BiLSTM-CRF and 1.53% over BERT-BiLSTM-CRF. In summary, the *F*_1_ score for each entity recognized by the new model are also improved significantly.

Based on the comparison of the above analysis, it can be seen that the model after the introduction of ALBERT is optimal in the recognition of DRN, COM and ADR, especially in the recognition of ADR with a much higher *F*_1_ score than the other models, which indicates that the inclusion of the ALBERT pre-training model in the input representation layer has a significant effect on improving the recognition of named entities.

There are still areas where this paper could be improved and future work could be done as follows.

(1) Improving the quality of data. In order to ensure the training sample size, the original data has “not yet clear,” so the data can be filtered to remove such data that aren’t relevant to the training of named entity recognition of adverse drug reactions, so as to improve the quality of the data. There are many statements with the same meaning and different expressions in the description of adverse drug reactions, in the data pre-processing stage, a similar word conversion module can be set up to convert such synonyms as " increase” and " raise” into a uniform expression.
(2) The self-built annotation dictionary is time-consuming and labor-intensive. Further, the annotation dictionary can be constructed by using word separation and lexical annotation methods, and multiple word separation and lexical annotation tools can be used to improve each other in order to increase the accuracy of the data. In addition, if there is an authoritative dictionary or corpus of adverse drug reactions, it can be considered as a dataset to experiment with the model again.

## 7 Conclusion

In this paper, we proposed an ALBERT-BiLSTM-CRF model-based named entity recognition method for adverse drug reactions, based on a manually constructed corpus for the recognition of three types of named entities: drug name, drug component and adverse drug reaction, and compared it experimentally with two classical models, BiLSTM-CRF and BERT-BiLSTM-CRF, respectively. The experimental results show that the method in this paper achieves an overall *F*_1_ of 91.19%, which is 1.5% and 1.37% better than the other two models respectively, and the performance of all three types of entities is significantly improved, which proves the superiority of the method proposed.

In the future, the model will also be considered to identify other entities of a drug, such as the former name of the drug, its English name, applicable symptoms, possible cross-reactions arising from the simultaneous use of two drugs, etc. In addition, based on the identification of named entities of adverse drug reactions, entity relationship extraction will be carried out so that the knowledge graph of adverse drug reactions can be constructed and be useful in practical applications such as intelligent diagnosis, risk inference and automatic question and answer.

## Data Availability

The datasets presented in this study can be found in online repositories. The names of the repository/repositories and accession number(s) can be found below: https://github.com/2193355137/NER-dataset.
